# The role of TLRs in cervical cancer with HPV infection: a review

**DOI:** 10.1038/sigtrans.2017.55

**Published:** 2017-11-03

**Authors:** Xiao Yang, Yanxiang Cheng, Chunsheng Li

**Affiliations:** 1 Department of Obstetrics and Gynecology, Renmin Hospital of Wuhan University, Wuhan, China; 2 Department of Obstetrics and Gynecology, University of Pennsylvania Perelman School of Medicine, Philadelphia, PA, USA

## Abstract

The main cause of cervical cancer is persistent infection with high-risk human papilloma virus (HR-HPV), but not all human papilloma virus (HPV) infections lead to cervical cancer. The key factors that determine the outcome of HPV infection remain poorly understood, and how the host immune system protects against HPV infection is unclear. Toll-like receptors (TLRs) are a group of pattern recognition receptors present in the cytoplasm and cell membrane, and can specifically recognize pathogen-associated molecular patterns. As the key molecules of innate and acquired immunity, TLRs not only play important roles in the immune defense against infectious diseases, but also are involved in the occurrence and development of a variety of malignant tumors. In cervical cancer caused by HR-HPV infection, TLRs have been found to regulate the local immune microenvironment. The role of TLRs in HR-HPV infection and HPV-induced cervical cancer and its relationship with HPV vaccine are reviewed in this article.

## Introduction

**Figure 1:**
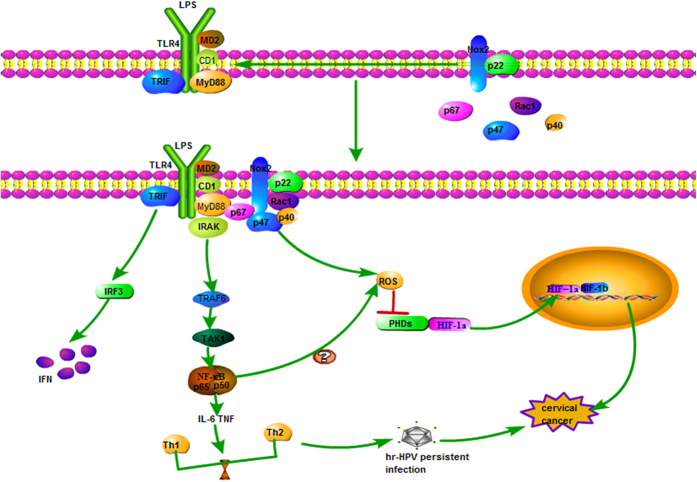
The possible mechanism of TLR4 being correlated with cervical cancer. The combination of TLR4 and its ligand (LPS) can trigger lipid rafts flowing which results in the change of lipid raft space conformation. This conformational change provides a condition for the aggregation of NADPH oxidase subunits on lipid rafts, which activates the redox reaction of lipid rafts to produce ROS and inhibits the degradation of HIF-1α, leading to the high expression of HIF-1α.

Cervical cancer is one of the most common gynecological malignancies. The incidence of cervical cancer ranks second among women, and is a serious threat to women's health.^1^ Nearly all cervical cancer is caused by high-risk human papilloma virus (HR-HPV) infection, mainly HPV16 and HPV18.^2^ In most cases of human papilloma virus (HPV) infection, the HPV virus can be cleared by the immune system. However, persistent HPV infection develops in 10–15% of cases, and about 1% of HR-HPV infections eventually develops into cervical cancer. Persistent viral infection can be associated with the ability of HR-HPV to escape immune clearance, but the exact mechanism is unclear. HPV viruses can interfere with the expression of Toll-like receptors (TLRs) and regulate TLRs signaling pathways to induce persistent infection, which in turn leads to cervical lesions and eventually to cervical cancer (Figure 1).

## TLRs

### TLR structure

TLRs are highly conserved between humans and mice. To date, 11 human TLRs and 13 murine TLRs have been identified.^3^ TLRs are composed of an extracellular region, transmembrane region, and intracellular region, which are typical for transmembrane receptors. The extracellular region consists of 10 to 30 leucine-rich repeats, which can identify the pathogenic microorganism’s pathogen-associated molecular patterns (PAMPs). The intracellular region contains approximately 200 amino acid residues, and has a high degree of homology with the cytoplasmic domain of interleukin-1 receptor (IL-1R). The cytoplasmic region plays an important role in downstream signaling.^4^

### TLR distribution

Based on subcellular localization, TLRs can be divided into two classes. One type localizes to the cell surface, and includes TLR1, 2, 4, 5 and 6, and mainly recognize the lipid composition of the surface of the pathogen. The other class of TLRs localize to the cytoplasm, and include TLR3, 7, 8 and 9, which mainly recognize the nucleic acid composition of the pathogen.^5^ TLRs are mainly expressed on innate immune cells, such as dendritic cells (DCs), mast cells, macrophages, neutrophils, endothelial cells and natural killer (NK) cells.^6^ In recent years, numerous studies have shown that TLRs are also expressed in tumor cells and cells in the tumor microenvironment of various cancers. In particular, TLR3, TLR4, TLR5 and TLR9 are frequently found in cervical cancer tissues.^7–10^ Furthermore, the activation of TLRs by different ligands plays important role in the development and progression of cervical cancer.

### TLR ligands

TLR ligand specificities are determined by the extracellular amino acid composition of the receptors. TLRs can also recognize endogenous signals from damaged cells and their degradation products, such as heparin sulfate, and high mobility group box 1 protein.^11^ TLRs and their ligands are shown in Table 1.

### TLR signal transduction pathways

**Figure 2:**
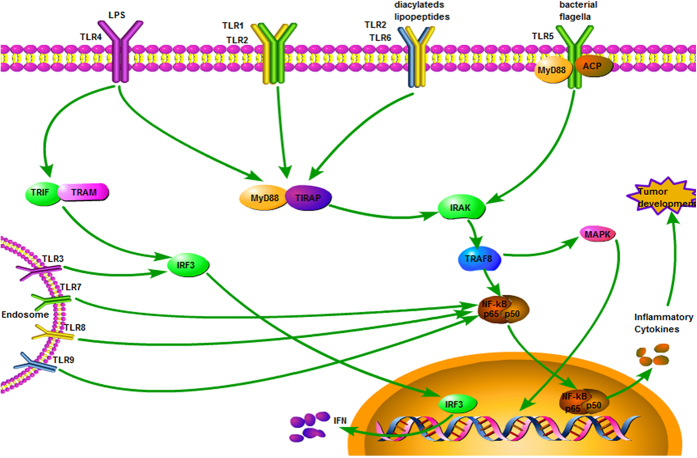
Toll-like receptor signaling. The MyD88-dependent pathway results in nuclear translocation of NF-κB and induction of pro-inflammatory cytokines. TLR3 and TLR4 activate IRF-3 and induce IFN-stimulated regulatory elements through a MyD88-independent signaling pathway.

At present, TLRs signal transduction pathways are divided into the human myeloid differentiation factor 88 (MyD88)-dependent pathway and MyD88-independent pathway. TLR1, TLR2, TLR5, TLR6, TLR7, TLR8 and TLR9 signals are mediated by MyD88-dependent pathway, and TLR3 signaling is by the MyD88-independent pathway, while TLR4 can activate and transduce signals through both pathways simultaneously.^12^ TLR3 and TLR4 can activate NF-κB in cells without MyD88.^13,14^ Also, TLR3 and TLR4 have been shown to activate IRF-3 and induce IFN-stimulated regulatory elements through a MyD88-independent signaling pathway.^15,16^ After binding to ligands, TLRs activate signal transduction cascades either via myeloid differentiation factor 88 (MyD88) pathway or the TIR joint protein molecules (TRIF) pathway, causing the activation of nuclear factor kappa B (NF-κB), mitogen activated protein kinase (MAPK), and other signaling pathways in a variety of immune cells. Eventually, activation of these pathways can induce the expression of a series of functional molecules, such as cytokines, trend factor and inflammatory factor, and initiate immune responses to enable natural defenses. There are at least three mechanisms in which TLR-regulated inflammatory response can promote carcinogenesis: via activating the anti-apoptotic effect of NF-κB, a transcription factor important in inflammatory conditions, via causing oxidative damage to DNA, and via inducing the tissue repair response^17^ (Figure 2).

## Relationship between TLRs And Cervical Tumor Immunity

### TLRs and immunity

**Figure 3:**
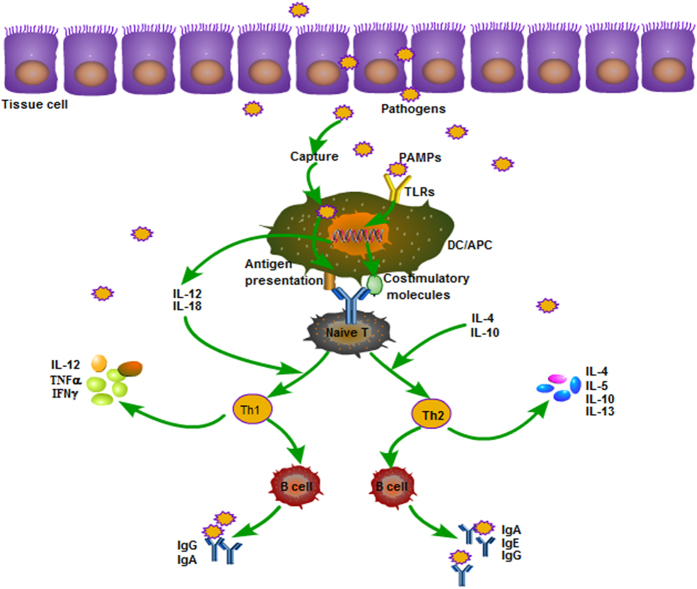
TLRs and T helper cell responses. PAMPs from invading pathogens bind with TLRs expressed in DCs, and DCs become activated and mature to active naive T cells. Naive T cells are primed toward specific T helper profiles: Th1, Th2, which can produce cytokines and in turn establish resistance to external pathogen invasion.

After recognizing microbial pathogens and other ligands, TLRs can induce DCs and macrophages to produce type 1 interferon and inflammatory cytokines. Activated TLRs can also induce DC maturation and enhance the antigen-presenting ability of DCs. TLRs can also regulate the proliferation and differentiation of Th1 cells and Th2 cells, which in turn establish resistance to external pathogen invasion. While most inflammatory mediators induced by TLRs can help to remove pathogens, excessive inflammatory mediators may lead to the occurrence of pathological inflammation, such as autoimmune disease, rheumatoid arthritis, inflammatory bowel disease and system lupus erythematosus.^18^ TLRs and T helper cell responses as shown in Figure 3. Macrophages and DCs can be inhibited via TNFAIP3, also termed tumor necrosis factor inducible protein A20, PTPN22, also termed LYP, or the BCAP/PI3K pathway. These three mechanisms can negatively regulate TLR-mediated immune response and influence the pathogenesis and treatment of autoimmune diseases.^19–21^ LYP in mononuclear cells can downregulate the activity of TLRs, and LYP inhibits TLR responses through the inhibition of the TREM2/DAP12 pathway.^22^ In aggregate, TLR activation can be a double-edged sword to the well-being of an organism. To maximize the benefit and harness the damage of TLR activation, greater efforts are required to understand the role of TLRs in immunity.

### TLRs and tumors

TLRs are important components of the human immune system. They play important roles in combating viruses, bacteria, and other infections, and function in anti-tumor immune activity. Some recent studies have found that TLRs could facilitate the initiation, progression, and metastasis of tumors. In recent years, more and more studies have confirmed that TLRs expressed in tumor tissues with different subtypes of TLRs, such as gastric cancer,^23,24^ colorectal cancer,^25,26^ ovarian cancer,^27^ lung cancer,^28^ prostate cancer,^29^ breast cancer,^30,31^ liver cancer^32,33^ and pancreatic cancer,^34^ which were actived by different ligands playing an important role in the occurrence and development of tumor. The expression of TLRs in different tumor tissues is shown in Table 2. Furthermore, tumor development often is associated with abnormal expression of TLRs and chronic inflammation, yet the exact mechanism is not clear. de Matos *et al.*^35^ found that the expression levels of TLR2 and TLR4 were higher in cervical cancer and premalignant lesions compared to normal controls. Studies also found that TLRs, especially TLR4, TLR5 and TLR9, are closely related to HPV infection and cervical cancer. Activation of TLR4 can promote the growth of ovarian cancer cells.^36^ The endogenous TLR4 activator S100A8/S100A9 could promote growth and metastasis of tumors.^37^ HPV16 infection will activate the innate immune system and initiate an inflammatory reaction in cervical epithelium, which not only identifies and clears the HPV virus but can also promote cervical intraepithelial neoplasia, ultimately leading to cancer. Hasan *et al.*^38^ indicated that the HPV16 E6 protein inhibited TLR9 transcriptional pathways, affecting the immune systems’ ability to recognize pathogens, thereby enabling virus to escape from immune surveillance. However, this phenomenon was not observed in the transfection of HPV18 E6 into cells,^38^ suggesting that different types of HR-HPV mediate different regulatory mechanisms on TLR expression in tumor development. Studies show that TLR3 mRNA was increased in cervical atypical squamous epithelium. TLR1 mRNA was decreased in cervical squamous cell carcinoma, yet the expression levels of TLR1, 2, 5, 6 and 9 were increased in parallel with the increase in the level of cervical lesions.^39^ This result suggests that there are specific temporal and spatial regulations of TLR expression in the different stages of lesion tissue and of cervical cancer development. However, the mechanism of how TLRs are involved in regulating the outcome of HR-HPV infection has not been clarified. The relationship between TLRs and HPV can be accurately studied by subdividing the type of HPV, the type of TLRs, the grade of cervical lesions, and the composition of the tissues.

### TLR4 and cervical cancer

In recent years, multiple studies showed that hypoxia is often present in solid tumors, and hypoxia plays a very important role in tumor occurrence, progression, malignant invasion, distant metastasis, and tumor resistance to radiotherapy and chemotherapy. Our previous studies showed that the expression of TLR4 in cervical cancer is related to the occurrence, development, and growth of cervical cancer cells. Uncontrolled growth of cervical cancer cells is closely related to the excessive activation of an important transcription factor, hypoxia-inducible factor-1α (HIF-1α). The cervical TLR4 signaling pathway is positively correlated with high expression of HIF-1α.^40^ There is a hypoxic microenvironment in tumor tissues, and this hypoxic microenvironment can induce high expression of HIF-1α. However, the expression of HIF-1α and major histocompatibility complex class I -related chain A/B (MICA/B) are negatively correlated in cancer tissues. MICA/B are rarely expressed by normal cells, however, they are expressed in a variety of malignant diseases. Thus, these observations indicate that hypoxia is associated with immune escape of tumors.^41^ Tannahill *et al.*^42^ found that the activation of TLR4 via LPS strongly increases the levels of the TCA cycle intermediate succinate which is known to stabilize HIF-1α. Perhaps in the context of cancer an endogenous danger signal can use a similar mechanism of action to stabilize HIF-1α.^42^ This mechanism of action must be explored to provide a theoretical basis for finding targets for anticancer therapy.

#### TLR4 and the iNOS signaling pathway in cervical cancer

**Figure 4:**
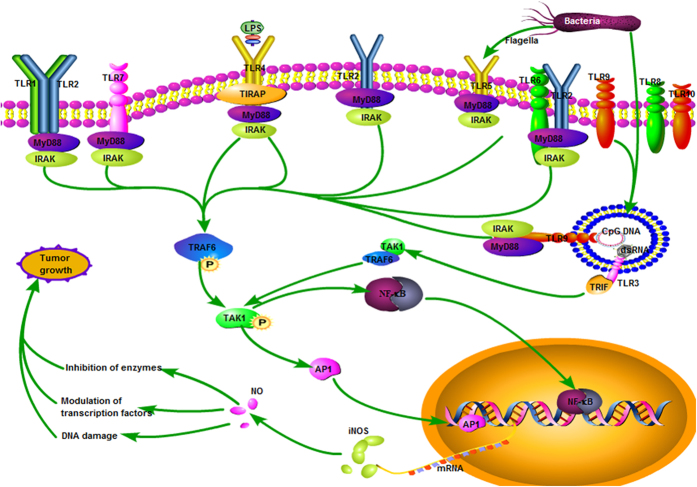
Synthesis of NO via TLR signaling. The TLRs are expressed on the cell surface or inside cells. TLRs use MyD-88 for downstream signaling, but TLR3 uses TRIF protein as an adaptor molecule. In downstream signaling, the activation of NF-κB or AP-1 can upregulate gene transcription for iNOS in the nucleus, and generate highly reactive NO, which can induce some effects and promote the proliferation of tumor.

TLR4 activates signal transduction by the MyD88-dependent and MyD88-independent pathways, which in turn modulate inducible nitric oxygen synthase (iNOS) through tumor necrosis factor receptor-associated factor 6 (TRAF6), MAPK, NF-κB and other key genes.^43^ Rahkola *et al.*^44^ found that patients with HR-HPV infection have higher nitric oxide (NO) in the cervical canal than do HPV-negative patients, indicating that the release of NO is closely related to HPV infection. The expression of iNOS in prostate cancer, head and neck squamous cell carcinoma, gynecological tumors,^45^ breast cancer, and lung cancer tissues was different from that of normal tissue. In prostate cancer, the expression of iNOS was related to tumor cell proliferation.^46^ In breast cancer, iNOS and NO can stimulate the expression of ERBB2 and bFGF and promote the proliferation of breast cancer cells.^47^ In cervical cancer, iNOS expression was positively correlated with tumor clinical stage, malignant degree, distant metastasis, and tumor microvessel density. When iNOS activity was inhibited, the growth of tumor cells was also inhibited.^48^ Xiao *et al.*^49^ detected the mRNA and protein expression of TLR/iNOS pathway transduction molecules including TLR4, NF-κBp65 and iNOS in different cervical cell lines, and they found TLR4/iNOS pathway is highly expressed in cervical cancer with hrHPV infection which may be resulted in cervical tumorigenesis. Synthesis of NO via TLR signaling as shown in Figure 4. However, the mechanisms of how the cervical local microenvironment regulate the expression of iNOS in cervical lesions of different stages, thereby affecting HR-HPV infection and disease prognosis, and how HR-HPV escapes the cytotoxicity of high concentrations of NO upon iNOS induction, remain unclear. The answers to the above questions will help us to further understand the process of immune clearance of HR-HPV and the HR-HPV carcinogenic mechanism.

#### TLR4 and the NF-κB signaling pathway and cervical cancer

In recent years, NF-κB has been the subject of intense research interest and has become an attractive drug target. The regulation of NF-κB activity in cells could treat certain inflammatory diseases and control tumor progression.^50^ NF-κB is downstream of TLR4 signaling, controlling expression of various cytokines and inflammatory factors. The TLR/MYD88/NF-κB signaling pathway plays an important role in inflammation, tumor development and invasion.^51^ Our previous studies showed that TLR4 expression was substantially elevated in cervical carcinoma.^40^ Moreover, high TLR4 expression further activated NF-κB regulated inflammatory signaling pathways, including the HIF-1α signal pathway that promoted the production of the immunosuppressive cytokines IL-6, TGF-β1, and others, which promoted the growth of Hela cells and enhanced their resistance to apoptosis.^50^ Xue-feng *et al.* showed that BPA induced migration of cervical cancer cells through the IKK-β/NF-κB pathway, presumably by upregulating expression of metalloproteinase-9 (MMP-9) and fibronectin (FN) in Hela cells and Siha cells. Inhibition of NF-κB could eliminate the BPA-mediated upregulation of MMP-9 and FN. However, there were no significant changes of PKA, ERK1/2, EGFR or PI3K/AKT.^52^ Thus further investigation of TLR4 and the NF-κB pathway and their mechanism of action will help provide new ideas in developing therapeutic strategies to treat cervical cancer.

### TLR9

TLR9 ligands include unmethylated deoxycytidyl-deoxyguanosin dinucleotide (CpG) motifs in bacterial and viral DNA and oligonucleotide CpG motifs. The TLR9 signaling pathway is a MyD88-dependent signal transduction pathway. TLR9 can initiate signal transduction by directly combining with CpG oligodeoxynucleotides (ODN) and increasing MyD88. This activation leads to the expression of endothelial adhesion molecules, inflammatory cytokines, interferon and co-stimulatory molecules, and plays an important role in the process of immunity, cell apoptosis, and anti-infection.^53^

Lee *et al.*^54^ found that compared with the normal cervical epithelium, the expression of TLR9 in cervical cancer was significantly increased. Cannella *et al.*^55^ found that TLR9 levels were much higher in patients persistently positive to the same HPV genotype, compared with women who cleared HPV infection and with those re-infected with a different genotype. This finding indicates that the increased TLR9 levels without HPV clearance in persistently infected women could drive inflammation, thereby contributing to cervical cancer risk. Jiang *et al.* showed that CpG ODN is a specific agonist of TLR9, activating the TLR9 pathway by upregulating transcription of TLR9 to play immunoregulatory effects.^56^ Di *et al.*^57^ found that using the specific ligand of TLR9, CpG ODN, to stimulate the A549 lung cancer cell line can produce resistance to apoptosis induced by TNF-α. Therefore, TLR9 is beneficial to the survival of tumor cells. However, the mechanism of the role of TLR9 in the development of cancer remains to be elucidated.

## Immune escape mechanism of HPV in cervical lesions

### Structure and function of HPV

Humans are the only host of HPV, which mainly infects the skin and mucosal epithelial cells, with viruses being divided into skin types and mucosal types. Bosch and Manos *et al.* detected cervical cancer tissues from 22 countries by PCR, they found that HPVDNA were expressed in 99.7% of the tumors with no significant difference between countries.Research also found, HPV16 type 18 infection rate was the highest in all types, HPV16 accounted for 50%, HPV18 accounted for 14%, HPV45 accounted for 8%, HPV31 accounted for 5%, other types of HPV accounted for 23%. The type of HPV was associated with the pathological type of cervical cancer. HPV16 occupied the main position in cervical squamous cell carcinoma (51% squamous cell carcinoma specimens), and HPV18 occupies the main position in the adenocarcinoma of the uterine cervix (56% specimens of glandular epithelial cell carcinoma) and cervical glandular squamous cell carcinoma (39% specimens of adenosquamous cell carcinoma).^58,59^ Multiple studies have confirmed that HPV is detected in 98.5% of invasive cervical cancer, suggesting that HPV infection is closely linked to cervical cancer.^60^

**Figure 5:**
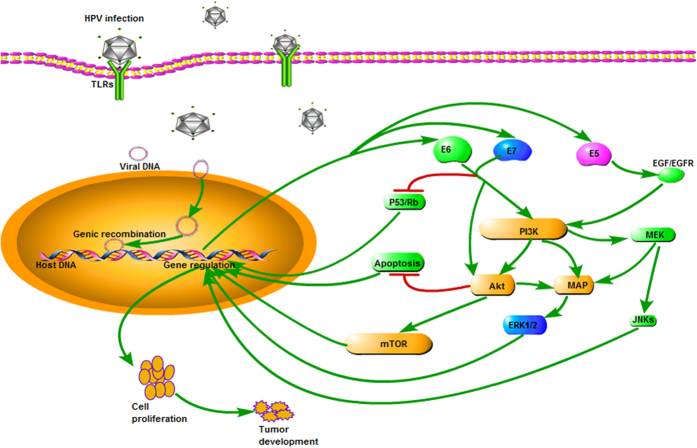
HPVs infect host epithelial cells through TLRs. The HPVs DNA viral replication is synchronous with host cellular DNA replication. The cells express E6/E7/E5 oncoproteins to damage the infected cells by inhibiting the expression of tumor suppressors p53 and Rb and decreasing apoptosis. E6/E7/E5 oncoproteins can also activate the PI3K/Akt/mTOR signaling pathway. All these processes enhance cell proliferation and promote tumor growth.

HPV is a circular double-stranded DNA virus with a non-enveloped icosahedral structure. A total of 100 subtypes of HPV, such as HPV16, HPV18, HPV32, HPV52, HPV58 and so on, have been identified, and 38 can infect the genital tract. Depending on the oncogenic ability, HPV can be divided into low-risk and high-risk types. Low-risk HPV mainly causes benign lesions, and HR-HPV, such as HPV16 and HPV18, can lead to malignant tumors.^61^ With persistent infection of HR-HPV in cervical epithelial cells, viral genes can be integrated into the host genome, resulting in squamous intraepithelial lesions through a cascading process. The HPV E2 gene encodes the early proteins which suppress the expression of E6 and E7. E6 and E7 proteins can inhibit the expression of tumor suppressor genes p53 and Rb, resulting in malignant transformation of the host cell and eventually the occurrence of cancer.^62^ HPVs infect host epithelial cells through TLRs as shown in Figure 5.

### The relationship between HPV infection and immune escape

Cervical cancer is characterized by persistent infection with HPV. In some cases, however, the HPV virus can escape from immune surveillance, the mechanism of which remained unclear until recently. The immune system is crucial for virus elimination. Macrophage, NK cells and T lymphocytes all play important roles as the first-line defenders in eliminating HPV infection. The second line of defense is cytotoxic T lymphocytes (CTLs) inducing an adaptive immune response against HPV16 E6 and E7 to eliminate HPV infection.^45^ However, HPV can escape immune attack through a variety of mechanisms. First, HPV does not kill host cells during viral replication, and therefore does not present viral antigen nor induce inflammation. HPV16 E6 and E7 proteins can downregulate the expression of type 1 interferons (IFN-1) in host cells. IFN regulatory factor-1 (IRF-1) can induce the production of antigen presentation transporter (TAP-1), IFN-β and monocyte chemotaxis protein-1 (MCP-1), which is very important for cellular immunity and plays a role in the prevention of inflammation in infected sites. The lack of co-stimulatory signals by inflammatory cytokines, including IFNs, during antigen recognition may induce immune tolerance rather than the appropriate responses. Moreover, HPV16 E5 protein downregulates the expression of HLA-class 1, and reduces recognition by CD8 T cells.^63^ The lack of cytokine production in HPV-infected sites prevents the activation and maturation of DCs, thus inhibiting the activation of a positive CTL response.^64^

At the same time, it was showed that HPV E5 and E7 proteins inhibited the expression of major histocompatibility complex class I proteins on the cell surface. HPV-infected cells are resistant to lysis by NK cells, but are sensitive to cytokine-activated NK cells. Activated macrophages play an important role in the elimination of HPV-infected cells and control the development of tissue malignancy.^65^ Therefore, innate immunity plays a key role in the prevention and elimination of HPV infection, but immune escape and inactivation of innate immunity still occur, which may eventually lead to persistent HPV infection, and further lead to the occurrence of cervical cancer.

### The relationship between HPVE6 protein and cervical cancer

HPV infection can lead to a series of biochemical changes, such as regulation of cell cycle, virus copy, apoptosis, cell proliferation and cell malignant phenotype. All of these can be regulated via the E6 and E7 proteins.^62^ The expression of the HPV16 E6 protein is closely related to the occurrence of tumors. E6 protein plays a role in HPV infection through a variety of mechanisms. It binds to P53 and accelerates the degradation and inactivation of that tumor suppressor, thereby inhibiting cell apoptosis and promoting the occurrence and development of tumors.^66^ It regulates the activity of telomerase, thereby inhibiting the normal process of cell apoptosis and promoting cell immortalization.^63^ It can also cooperate with the death domain–associated protein (DAXX) to inhibit apoptosis.^67^ The HPV16 E6 protein also interacts with IFN-3 to downregulate the expression of IFN-α and IFN-β; it inhibits the antiviral activity of IFNs and downregulates TNF-α and IL-1β secreted by macrophages following activation by LPS, both of which help tumor cells escape from the innate immune response of the host.^68^ HPV16 E6 protein is closely related to the pathogenesis of cervical cancer, so it is very important to understand the biological function of E6 and to study its effect on cervical cancer and its mechanism.

## Correlation between hpv escape from immune clearance and TLRs

Chronic inflammation is closely related to tumor development and progression.^69^ However, the tumorigenic mechanism of chronic inflammation is unclear. Xiao *et al.* initially explored the expression of the TLR/NO pathway in HR-HPV-positive cervical cancer tissues. They found that the key genes of TLR4/NO pathway, such as TLR4, NF-κBp65, and iNOS, are highly expressed in HR-HPV-positive cervical cancer tissues and cells,^49^ strongly suggesting that the TLR4/NO signal pathway may be associated with the HR-HPV infection. This pathway may mediate alterations of the local immune microenvironment in cervix, facilitating immune escape or immune tolerance of tumors, eventually leading to the occurrence, development, and metastasis of cervical cancer. Specific mechanisms of the TLR4/NO pathway and HR-HPV involved in cervical cancer need further exploration.

Intriguingly, some studies revealed that the NO content with HR-HPV infection was significantly higher than that of low-risk HPV infection and no HPV infection, thus the NO content of the cervical canal was closely related to the HR-HPV infection status.^70^ Daud *et al.* found that cervical epithelial cells with low expression of TLR9 were more susceptible to HPV infection, and cervical epithelial cells with high TLR9 expression rarely have any HPV infection, suggesting that the elimination of HPV infection may involve the increased expression of TLR9.^71^ Hasan *et al.* showed that HPVE6 protein can downregulate the expression of the TLR9 gene and inhibit the expression of TLR9 signaling pathway related molecules, thus affecting identification of pathogens by the body and facilitating escape of HPV infection from the immune system. Hasimu *et al.*^72^ found that TLR4, 7 and 9 expression changes significantly in cervical cancer tissue. In addition, the expression levels of TLR4 and TLR9 were positively correlated with HPV16 infection. Altogether, these findings suggested that TLR4 and TLR9 may be the receptors for HPV infection. However, the exact roles of TLR4 and TLR9 in immune escape and the key molecular pathways involved remain unclear, and further study will be needed.

HPV can escape from TLR to invade the innate immune system. The HPV16 E6 protein can enhance the expression of functional components of the NF-κB signaling pathway, including p50, NIK and TRAF-interacting protein, and increase their binding to NF-κB and AP-1 DNA consensus binding sites. The studies of Lee *et al.* suggested that the expression levels of TLR9 gradually increased from cervical dysplasia to invasive cervical cancer tissues. However, the study did not further clarify the relationship between the increased TLR9 and cancer.^73^ Yu *et al.*^74^ thought that the progression of cervical cancer was related to TLR4 due to frequent exposure to a variety of bacteria. However, they also observed that TLR4 expression declined during the progression of cervical cancer, and this downregulation of TLR4 appeared to be associated with the expression of P16INK4A, which is a crucial marker of HPV integration into host cells.^74^ These data may offer further insight regarding the association of HPV infection and TLR signaling during the development of cervical cancer.

However, our previous study found that TLR4 trended upward with increased disease severity after HPV infection, and the difference was statistically significant.^40^ The expression of HPV16 E7 in cervical lesions positively correlated with the severity of the lesion, but the difference was not significant. The correlation analysis showed that the expression of TLR4 in cervical cancer is irrelevant to HPV16 E7 (*r*=0.121, *P*=0.0612). Our results also showed that the occurrence and development of cervical cancer were associated with the expression of TLR4 in HPV16-infected cervical lesions, but the severity of cervical lesions did not necessarily correlate with gene expression. This observation suggests that cytokine production regulated by other pathogens mediated by TLR4 may be involved indirectly in the process of persistent HPV infection, and that the immune response was synergistic with HPV infection through the TLR4 pathway to lead to tumor development. Since the high expression of TLR4 is closely linked with the development of cervical cancer but is not mediated by HPV, we reasoned that other pathogens, such as bacteria, may activate the TLR4 pathway to facilitate immune escape, which promotes the development of cervical lesions. However, the relationship between TLR4 and HPV infection, and whether the inhibition of TLR4 signaling pathway could inhibit cervical lesions after HPV infection, requires further study.

## HPV vaccines and TLR agonists as an adjuvant in treatment of cancer

HPV infection is currently the only clear cause of cervical cancer. The HPV prophylactic vaccine is a major breakthrough for cervical cancer prevention. However, it still faces some challenges in clinical practice. At present, improving the efficiency of the HPV vaccine is an area of active investigation. The HPV therapeutic vaccine aims to reduce or eliminate tumor cells infected with HPV through cell immunity and humoral immunity and to prevent precancerous lesions from continuing to deteriorate. However, a therapeutic vaccine was disappointing, demonstrating severe adverse reactions and having difficulty in recognizing tumor-specific target antigens.^75^ The persistent expression of HPV16 E6 and E7 protein is essential for tumor cell transformation and maintenance of malignancy in most cervical cancers. Moreover, precancerous lesions associated with HPV16 E6 and E7 protein can stimulate anti-tumor Cytotoxic T lymphocyte (CTL)–mediated cytotoxicity. Therefore the E6 and E7 protein are seen as tumor-specific antigens and are the focus of research and development of an HPV therapeutic vaccine. Many groups, including us, developed HPV16 E6 monoclonal antibodies, which provide a convenient tool for the quick detection of E6 protein.^76^ Furthermore, E6 protein structure-function analysis can clarify its role in cervical cancer development and facilitate preparation of a therapeutic HPV vaccine.

Gardasil and Cervarix are considered prophylactic cancer vaccines, as they have been developed to prevent infection with HPV serotypes that are associated with almost 70% of cervical cancer cases.^75^ Rosenberg *et al.*^77^ reported that the objective response rate was low (2.6%) in a clinical trial with 440 cancer patients, with a lack of powerful adjuvants capable of overcoming the immunosuppression present in cancer patients. Thus, adjuvants are required to induce potent and durable immune response.

Molecules that can activate TLRs may be utilized for vaccine development and act as vaccine adjuvants. At present, the research and development of novel vaccine adjuvants are mainly focused on TLR ligands. Targeting TLR signaling pathways has been applied in clinical practice to improve the immunogenicity of DNA vaccines and promote the adjustment of T cells in resisting viral infection or to inhibit the wide spread inflammatory response caused by bacterial infection.^78^ Investigations showed that simultaneous activation of multiple pathways of TLRs by vaccines resulted in better immunogenicity effects. The high immunogenicity of the yellow fever vaccine is related to activation of multiple TLR signaling pathways in DCs. Activation of TLR signaling pathways can not only induce inflammatory reactions, but also can promote DC maturation and Th cell differentiation and produce an acquired immune response. Activation of TLR signaling pathways can be found not only in the immune response mechanism of the HPV vaccine, but also immune response mechanism of the rubella, measles, and hepatitis B vaccines.^79^ Vaccines and natural pathogens, in activating the pathogenic molecular activation pattern recognition receptor (PRR) pathway, induce innate immunity and are captured by DCs or other antigen-presenting cells in immune organs. The antigens are presented to B and T lymphocytes and produce an acquired immune response.

The distribution of different subtypes of TLRs differs among cells. Many studies found an important role of TLR pathways in infectious diseases and identified therapeutic advantages of novel agonists and antagonist ligands targeting TLRs. Hedayat *et al.* investigated the potential application of TLR ligands as vaccines or drugs. Modulators of TLR signal processing have been applied in clinical practice to improve vaccine effectiveness and promote Th1-type immune responses in viral or bacterial infection.^77^

Presently, only three TLR agonists are approved by international regulatory agencies for use in cancer patients: monophosphoryl lipid A (MPL),^80^ bacillus Calmette-Guérin (BCG), and imiquimod.^81,82^ MPL is a TLR4 ligand. Similar to LPS, it can activate the TRAM and TRIF signaling pathways and also can reduce the MYD-88 dependent signal pathway significantly that promoting inflammation.^80^

TLR binding of ligand can promote the differentiation and maturation of dendritic cells. It can stimulate the expression of molecules on the surface of the antigen-presenting cells and promote the production of cytokines. TLR signaling can also stimulate the transformation of naïve T cells into regulatory T cells and enhance the long-term protection of the host immune system.^83^ Pre-clinical trials in mice showed that glucopyranosyl lipid A (GLA) can function as a strong Th1 adjuvant that can also stimulate potent cellular immune responses.^84^ However, the only TLR4 agonist approved for human application is monophosphoryl lipid A (MPL), which has been tested in many clinical trials as a cancer vaccine adjuvant.^85^

Adjuvant System 04 (AS04) combines MPL and aluminum salt. A study finds that AS04-induced innate responses are primarily due to MPL, and aluminum can extend the cytokine responses to MPL. AS04 enhances vaccine response by rapidly triggering a local cytokine response, leading to an optimal activation of antigen-presenting cells.^86^ A clinical trial of the Cervarix vaccine with aluminum-MPL adjuvant and of the Gardasil vaccine with aluminum shows that MPL can enhance the effectiveness of the vaccine. The trial also shows that induced neutralization antibody titers were higher with Cervarix plus aluminum-MPL than with Gardasil plus aluminum.^87^ Compared with Gardasil, memory B cells in the blood increased significantly after HPV16/18 serum negative women receiving three immunizations with Cervarix.^88^

Currently, some clinical trials have indicated that vaccines administered with MPL as an adjuvant are safe and immunogenic.^89^ Moreover, the TLR7 agonist, TMX-101 (Vesimune), is currently being tested as an immunotherapeutic agent in a phase II clinical trial for noninvasive bladder cancer patients.^90^ Furthermore, several human clinical trials evaluating the adjuvant activities of CpG ODNs demonstrated that CpG ODNs can induce Th1-type immune responses, thereby becoming potential cancer vaccine adjuvants. Clinical trials using CpG ODNs as immunotherapeutic agents in cancer patients suggested that CpG ODN as monotherapy or in combination with chemotherapy can induce potent anti-tumor immune responses that correlate with clinical benefit.^91,92^ However, the potency of CpG ODN as a cancer vaccine adjuvant or anti-tumor agent needs further investigation.

Recently, adjuvant systems using different combinations of TLR adjuvants, including alum, MPL, and CpG ODN, have shown better efficacy compared with a single TLR adjuvant,^93^ suggesting that identifying optimal combinations of current adjuvants may be a better means of developing novel adjuvants for cervical cancer immunotherapy.

## Conclusion

The control of cervical cancer includes the prevention of primary HPV infection by vaccination and the detection and treatment of cervical precancerous lesions. HPV infection and immune escape lead to persistent HPV infection and eventually to cervical cancer. However, the immune escape mechanism of HPV infection is very complex, involving a variety of cellular and molecular interactions, and needs further investigation. TLRs play extremely important roles in innate and acquired immunity and are also key regulatory factors in tumor development.

At present, many signaling pathways are under intensive investigation, but there is no clear mechanism. Although studies have shown that TLRs have changed cervical cancer infected with HPV, the significance of this change and whether HPV infection can provide a new target for the treatment of cervical cancer still requires further study. Some studies also showed that there may be other pathogens that promote cervical lesions after HPV infection, and these may be coordinated with the TLR4 signaling pathway. TLR4-induced HIF-1α may be involved in tumor immune escape as well, providing a new concept for the further analysis of HPV pathogenesis. The balance between virus and immune factors provides key information for HPV-associated cervical cancer prevention and treatment with vaccines. Our team prepared an E6 protein monoclonal antibody for the preparation of therapeutic HPV vaccines. TLRs as molecular adjuvants provide a new target for HPV infection prevention and provide direction for development of efficient vaccines. Since TLRs are of great significance in the response to HPV vaccines, further studies on the TLR family are warranted.

## Figures and Tables

**Table 1:** TLRs and their ligands

*TLRs*	*Ligands*
TLR1/2	Triacylated lipopeptides^94^
TLR2	Mycoplasma, non-lipopeptidic PAMPs from various pathogens^94^
TLR2/6	Diacylated lipopeptides^94^
TLR3/7/8	Viruses or bacteria mainly in the nucleic acid^95^
TLR4	Lipopolysaccharide (LPS) of gram-negative bacteria^94^
TLR5	Bacterial flagella^96^
TLR9	Unmethylated DNA of bacteria or virus^97^
TLR10	Remain unidentified
TLR11	*T. gondii* profilin and uropathogenic *Escherichia coli*^98^

Abbreviations: PAMP, pathogen-associated molecular pattern; TLR, Toll-like receptors.

**Table 2:** The expression of TLRs in different tumor tissues

*Tumor tissues*	*Expression of TLRs*
gastric cancer	TLR2, TLR4, TLR5, TLR9
colorectal cancer	TLR2, TLR3, TLR4, TLR5, TLR9
ovarian cancer	TLR2, TLR3, TLR4, TLR5
lung cancer	TLR2, TLR3, TLR4, TLR9
prostate cancer	TLR4, TLR9
breast cancer	TLR2, TLR3, TLR4, TLR9
liver cancer	TLR2, TLR3, TLR4, TLR6, TLR9
pancreatic cancer	TLR2, TLR4, TLR9

Abbreviation: TLR, Toll-like receptors.
